# Supplementary material for: Rapid and sound assessment of well-being within a multi-dimensional approach: The Well-being Numerical Rating Scales (WB-NRSs)
Source: PLoS One. 2021 Jun 14;16(6):e0252709. doi: 10.1371/journal.pone.0252709 (PMC8202918; doi:10.1371/journal.pone.0252709)
Supplement: S1 Appendix — (DOCX) [file pone.0252709.s010.docx]

**S1 Appendix**

*Well-being Numerical Rating Scales* (WB-NRSs)

| **Item** | ***English version:*** Please indicate the degree of wellbeing you currently experience in each of the four areas indicated below. We also ask you to indicate the degree of overall wellbeing you perceive at this precise moment. You can answer using the following scale from 1 to 10 where 1 indicates a state of *absolute distress* and 10 a state of *complete well-being*. | ***Italian Version***: La preghiamo di indicare il grado di benessere che nel momento attuale sperimenta in ciascuno dei quattro ambiti sotto indicati. Le chiediamo poi di indicare il grado di benessere complessivo da lei attualmente percepito. Può fare una crocetta su uno dei numeri compresi tra 1 e 10 dove 1 indica uno stato di *assoluto malessere* e 10 uno stato di *completo benessere*. |
| --- | --- | --- |
| **1** | Physical well-being | Benessere fisico |
| **2** | Psychological well-being | Benessere psicologico |
| **3** | Relational well-being | Benessere nelle relazioni con gli altri |
| **4** | Spiritual well-being | Benessere spirituale |
| **5** | General well-being | Benessere complessivo |
