# Supplementary material for: Rapid and sound assessment of well-being within a multi-dimensional approach: The Well-being Numerical Rating Scales (WB-NRSs)
Source: PLoS One. 2021 Jun 14;16(6):e0252709. doi: 10.1371/journal.pone.0252709 (PMC8202918; doi:10.1371/journal.pone.0252709)
Supplement: Attachment — Submitted filename: Response to reviews.docx [file pone.0252709.s011.docx]

PONE-D-21-01391

Rapid and sound assessment of well-being within a multi-dimensional approach: The Well-being Numerical Rating Scales (WB-NRSs)

Dear Dr. Gremigni,

Thank you for your letter dated April 7, 2021. We were pleased to hear that our manuscript was positively reviewed and is being considered for publication in *PlosOne*, subject to minor revision and response to the comments raised by the editor and the reviewers.

We thank both you and the reviewers for the helpful comments and suggestions and have modified the manuscript accordingly. We enclosed a copy of the revised manuscript as well as our point-by-point responses to the comments made by the reviewer. In the revised manuscript, we track all changes made to the earlier version.

Finally, we would like to take this opportunity to express our gratitude to Reviewer #2 for the sincere appreciation of our work.

Kind regards,

Francesca Chiesi

Corresponding Author of the Manuscript

--------

First of all, we confirm that a minimal portion of data presented in manuscript was peer-reviewed and formally published elsewhere, but we are firmly convinced that it does not constitute a dual publication and it should be included in the current manuscript. Indeed, the pre-post data for the present study was utilized for a different purpose — to provide evidence of the responsivity psychometric property of the scale (never mentioned in the previous papers) —and we employed a different approach for the analyses. Specifically, Bayesian analyses, which allowed quantification of evidence in favor of the alternative and null hypothesis, were used to reinforce and verify the significance of the pre-post differences.

***Reviewer #1:***

**Comment 1:** Two different goals are presented. It is advisable to unify the presentation of the aim.

**Response:** Thank you to the reviewer for pointing this out. We have modified accordingly.

**Comment 2:** I would suggest clarifying that the proposed interventions are based on the literature, which attests to their effectiveness. Such reinforcement would be a clarification indicating the effectiveness of the proposed measure, as the intervention must bring about such a change.

**Response:** Thank you to the reviewer for asking for further clarification. We specified that both the interventions were original strategies (using music and artistic features). Thus, there is not a specific literature to refer to. Provided that the intervention’s aim was to improve participants well-being, the effectiveness of the proposed measure in detecting such a change can be considered an important feature of the instrument.

**Comment 3**: In Study 1 clarify the use of such a heterogeneous sample. And how this choice of sample could be positive for the study.

**Response:** Thank you to the reviewer for asking for further clarification. We specified that the sample allowed us to have a representative sample of the population by age and gender, and that including clinical participants allowed us to test the effectiveness of the scale in the health domain where it is especially important consider psychosocial well-being while treating disease. As such, the scale that was developed is not only reliable and valid, but may also be generalizable to a variety of age groups and clinical and general population settings.

**Comment** **4**: Who was the convenience group? Does it represent the cognitive capacity of the target sample of the Scale?

**Response:** Thank you to the reviewer for this suggestion. The cognitive capacity of the students were not assessed as part of data collection. The sample was a convenience sample because they were university students part of a psychology course. Provided that they were admitted into university, the authors could speculate that they were of normal or above average in terms of cognitive capacity compared to the general population. We also believe that the task at hand (i.e., filling out a questionnaire where the well-being measure is a short descriptor) would be manageable for a wide range of individuals of different ranges of cognitive capacity and reading levels.

**Comment** **5**: In study 1 - Design and procedure - indicate when it was carried out the data collection. Having been in the period of the pandemic, if there was a need to change the form of the collection.

**Response:** Thank you to the reviewer for asking this clarification. Data were collected before the pandemic, specifically from January 2017 to April 2019.

**Comment 6**: Whereas the scales have different scores. Was a procedure used to standardize the score to ensure compatibility between the measurements? Especially in the discriminant validity? **Response:** Thank you to the reviewer for allowing to clarify this point. Since we performed correlational analyses, standardization was not required given that the correlation is normalized by standard deviation.

**Comment 7**: Could the absence of a common measure (in addition to the WB-NRS) in the sample subgroups hinder the analysis of discriminant validities?

**Response:** Thank you to the reviewer for allowing to clarify this point. As stated in Study 1 Analysis Strategy, Discriminant validity was tested to demonstrating that a measure does not correlate too strongly with measures they are not intended to. Thus, correlations between couples of variables were performed. We thank you for this suggestion that might be tested in future studies.

**Comment** **8**: Has the normality of the distribution of data for statistical tests that require this premise been guaranteed?

**Response:** Thank you to the reviewer for asking for this specification. The W-BNRSs scores were normally distributed (see kurtosis and skewness indices in Table 1 and Table 6). Now, we added that these indices fall within this range for all the variables in the study.

**Comment** **9**: Study 2 – row 532 - indicate when it was carried out the data collection, and the collection server (e.g. googleforms, surveymonkey…)

**Response:** Thank you to the reviewer for asking for this specification. We added the names of the servers.

**Comment 10** - It is suggested to update the references.

**Response:** References were updated.

***Reviewer #2:***

First of all, we would like to thank you and express our gratitude for the appreciation of our work.

**Comment:** Demographic variables (countries) are not presented in Study one, why?

**Response:** Thank you to the reviewer for pointing this out. Study 1 was conducted with Italian participants.

**Comment:** Factor loadings for Canadian are higher than Italian? Please, give interpretation for this result? Factor loadings discovered difference between two nations, while differential item functioning confirmed similarities. Please why results are discrepant? Please interpret?

**Response:** Thank you to the reviewer for pointing this out. Following this suggestion, we conducted a multigroup CFA. Results indicated that Metric invariance holds, i.e., measurement weights did not lead to a significant decrement in model fit compared with the configural model when constrained to be equal (ΔChi2= 6.91, *p*=.141; ΔCFI = .002; ΔRMSEA= .005). These results suggest metric invariance can be established and in line with IRT results, the scale is invariant across these two groups. Given that IRT does cover differential item functioning, we prefer not to add further analyses because we deem that IRT can be sufficient.
